# Explanation to Mr. Woodham, and Additional Remarks upon Vaccination

**Published:** 1815-01

**Authors:** Richard Walker

**Affiliations:** Oxford


*For the Medical and Physical Journal.*

*Explanation to Mr. Woodham, and additional Remarks upon Vaccination;* by Mr. R. Walker.

IN the last Number of the Medical and Physical Journal, I observed a communication entitled "Upon the Folly and Danger of Hypotheses, with remarks on Mr. Walker's innate Principle of Small-pox, by Mr. Woodham," in which that gentleman declares his "strong dislike" to hypotheses in general, and then proceeds to comment, under a similar impression, upon an opinion advanced by me, respecting small-pox and vaccination, in the paper to which the title alludes.

Had this gentleman passed over this hypothesis, as he considers it, he would hereafter have found, that I am as adverse to any preconceived theories or hypotheses as himself or any other person can be.

With respect to the quotations this gentleman has made respecting my opinions, in this instance, and his remarks upon them, I shall pass over them all, one excepted, being well aware that such opinions of mine as are there given, should they be ill-founded, cannot be productive of any evil tendency in practice. The circumstance alone which I shall notice is the following observation, produced in the manner of analogy, thus, "As well might it be said that this essence or innate principle is an alkali; and that the exciting cause is an acid, that coming in contact, an effervescence or commotion motion takes place in the lymph or blood, and a neutral salt is formed, which, being expelled from the system, leaves the person ever after secure from the disease."

Without remarking upon this supposed analogy, which is by no means congenial with my ideas of analogies, were I to attempt to elucidate my opinion respecting a prior existence in the system of the essence, or an innate principle of small-pox, its excitement into action, and its expulsion, by a chemical analogy, I should adduce the example of what happens in the instance of fermenting liquors, as beer, &c. The essence or innate principle of fermenting matter, as we well know, exists naturally in the beer; but, in order to expel or drive out this essence or innate principle, remaining dormant or latent, and from which it is necessary it should be purified by art, a small portion of the same essence, in the form of yeast, which had been previously produced by a similar process, is added—the desired ferment or commotion is excited, the original principle of fermentation existing in the beer is collected together, and finally expelled, or driven out, and used for a future similar purpose, and so on *ad infinitum.**

I must confess I consider this as an intelligible and tolerably apt illustration of my opinion. I have, however, to thank this gentleman (Mr. Woodham) for furnishing me with the hint of illustrating my opinion by a chemical analogy.

Being called back, as it were, to the subject of vaccination, I shall take the opportunity of reverting to a circumstance of no small importance respecting the practice of it.

Many practitioners are in the habit of making two punctures in vaccination, by way of ensuring success, and likewise for the purpose of transferring the affection to others.

With respect to the first reason for the necessity of more

---

* I have taken *malt liquor* as an example, because it is the most familiar instance of fermentation. The essence or innate principle in this instance I consider to be existing originally in the wort, viz. carbon and oxygen, the union of which, forming carbonic acid, is the natural or inherent cause of fermentation, which produces and throws out what is called yeast; and which is commonly used as a ferment, for the purpose of promoting fermentation in future processes of the same nature. Here then we have, by analogy, I think, a solution of this gentleman's supposed paradox or inconsistency, viz. "It is, therefore, according to circumstances, both agent and patient, cause and effect, the excitement to action, and the recipient of that excitement:" and this I take to be the case likewise in inoculated small-pox, and in vaccination.

than one puncture, I have already given my reason for dissenting from it. It is, however, asserted, that, if the pustule or vesicle be opened, and lymph or fluid taken out of it, the due progress of the pustule may be interrupted thereby, and the intention of security from small-pox by vaccination defeated.

Hence arises, it is stated, the propriety or rather the necessity of more than one puncture, reserving one untouched, or perfect, for the security of the patient, and the other for propagating the infection to others.

It is true, if the vesicle be opened for the purpose of transferring fluid to another person, so soon as it may admit of, viz. before the seventh or eighth day after vaccination, such a circumstance might happen: or if, when the vesicle is become full of fluid or lymph, it be then opened and drained entirely of its fluid, as is occasionally done by lint, or otherwise, in order to collect matter by wholesale for vaccinating a great number of persons from one, such a circumstance may possibly follow, as it likewise might in inoculated small-pox, and the above precaution then be necessary.

Under ordinary circumstances, however, as I am satisfied by my own experience, viz. where the matter is taken by the lancet only, however often repeated from a mature or full vesicle, viz. on the eighth day, or about that period, no such circumstance need be apprehended.

Hence the necessity of more than one puncture arises, not from any defect in the principle of vaccination, any more than in the instance of inoculated small-pox, but from the extraordinary demand for it, and the confined nature of the source from which it is procured, a circumstance I have noticed before, and the probable ill consequences which might arise from it.

If, as is pretended by some persons, the mere accidental rupture of the vesicle, discharging a portion of the fluid, were sufficient to interrupt the due progress and effect of such vesicle, and the security from small-pox be thus prevented, confidence in vaccination would be extremely dangerous, since a restless or irritable child might thus destroy or defeat the effect intended by vaccination, however many punctures had been made.

To the preceding observations on vaccination, it may not be improper to subjoin the following precaution.

Since it is an unquestionable fact, that although, ordinarily speaking, vaccination, properly passed through, renders such persons secure from small-pox throughout life afterwards, yet some anomalies or rare instances do occasionally occur,

 of of persons, in whom there is no reason to suppose there has been any defect or imperfection in the progress of vaccination, who afterwards, ordinarily, at some distant period of time, viz. about ten years, less or more, according to circumstances, have been attacked by small-pox.

Although the virulence of succeeding small-pox in such persons appears to have been so much ameliorated by the preceding vaccination, as to dissipate all apprehension of danger, or of creating any necessary alarm; yet it might, nevertheless, be prudent, especially in such persons whose apparent habit of body might indicate a more than ordinary disposition for receiving infection, viz. a peculiar fullness or impurity of habit, to repeat the operation of vaccination, provided such persons are liable to be exposed to infection from small-pox, which might afford a test of their security, or have the effect of eradicating such disposition, should any still exist in the habit.

Moreover, should such danger of infection present itself, and no vaccine fluid to be obtained, it might be justifiable to inoculate with small-pox, under the usual precautions respecting regimen, &c.

We sometimes hear of an instance, one I know of myself, in whom repeated vaccination has failed to produce the intended affection: this need be no matter of surprise, when it is recollected the same circumstance has happened in inoculation for small-pox; and that some persons have resisted the infection of small-pox, in the natural way, throughout life, although frequently exposed to it. In such a case, however, it would be prudent to vaccinate again at a distant period, or to inoculate.

Since nothing should be omitted which can tend to the security of individuals, and with a view to exterminate or annihilate small-pox, it might be advisable, at the distance of ten years or more, to vaccinate a second time, although no immediate danger of variolous infection might be present.

The Surgical Editor of the Medical Journal, in a note at p. 475, respecting my paper on the removal of enlarged tonsils, by an improved mode of applying the ligature, suggests, that the method by excision with curved scissars, which he has practised, is preferable to the method by ligature. Not having had any experience in this mode myself, although I believe it to be in use with some practitioners, I can say nothing as to the merits of it, the method by ligature only being what I have been accustomed to, and which I understand is still the most prevalent or general mode in practice.

Moreover, the same gentleman mentions an instance of a young young woman, whom he cured by excision, who had been dismissed the Oxford Infirmary, after an unsuccessful attempt by the method originally proposed by Cheselden, but which, I believe, is at present but little practised, viz. by conducting a double ligature through the base of the tonsil, and then including each half of the tonsil, by means of the two ligatures.

With respect to this circumstance, having had no communication with the Infirmary for seven or eight years past, I can only say confidently, that no such operation was performed, or attempted, during my residence there, to my knowledge.

The mode I have recommended for applying the ligature for the extirpation of enlarged tonsils, may be used with equal facility, I presume, in all tumours which require such an operation, when situated beyond the reach or command of the hand itself for performing it. I must confess, I think myself there is an eradicating effect produced, in consequence of the part sloughing away, when the ligature is duly applied, in extirpating enlarged tonsils, and such like enlargements, which may not take place by simple excision.

Since my communication respecting loose fractures, another case of the same kind has presented to me, in which I have applied vesicatories with my former success. In this instance, (an oblique fracture of the leg,) to which I was called in, seven or eight weeks after the accident, the leg is so perfect, and the bone so smooth, notwithstanding the frequent necessity of moving the limb, in consequence of the repeated application of vesicatories, that neither the eye nor finger can ascertain where the fracture was—in the former instances, fifteen or sixteen weeks had elapsed before the application of blisters. I think from three to five blisters may ordinarily be sufficient.

*Oxford; Dec.* 6, 1814. RICHARD WALKER.

---

*For the Medical and Physical Journal.*

